# Supplementary material for: Saccharomyces boulardii (CNCM I-745) improves intestinal damage in sepsis by remodeling intestinal flora structure
Source: Front Cell Infect Microbiol. 2025 Jul 31;15:1602792. doi: 10.3389/fcimb.2025.1602792 (PMC12350329; doi:10.3389/fcimb.2025.1602792)
Supplement: Supplementary file 1 [file DataSheet1.zip › summary/summary/8_Advanced_analyse/2_PICRUSt2_result/METACYC/Group.BW0_vs_AW0/PICRUSt2_METACYC_Group.BW0_vs_AW0_diff.pdf]

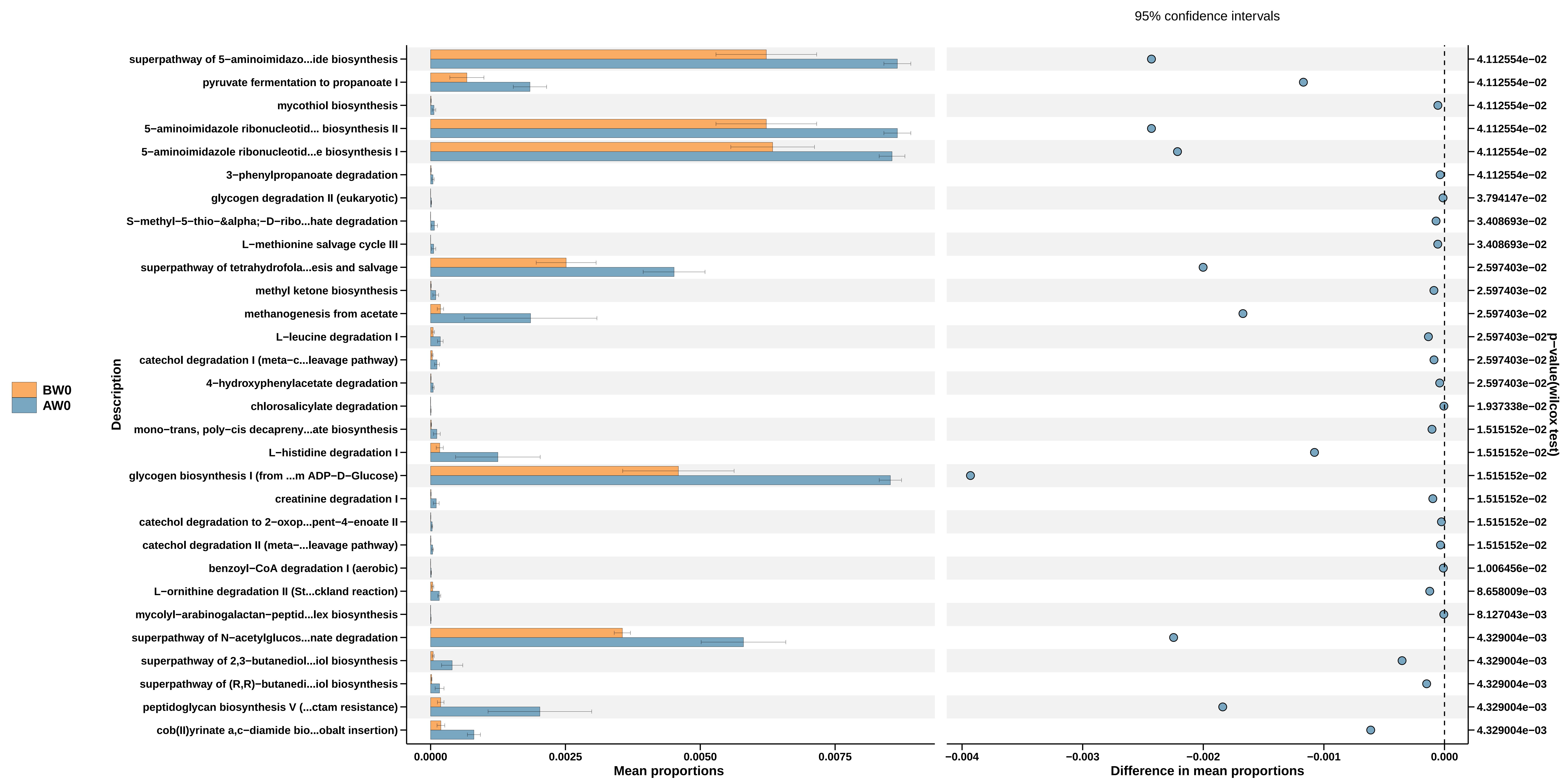

95% confidence intervals
BW0
AW0
Description
superpathway of 5−aminoimidazo...ide biosynthesis
pyruvate fermentation to propanoate I
mycothiol biosynthesis
5−aminoimidazole ribonucleotid... biosynthesis II
5−aminoimidazole ribonucleotid...e biosynthesis I
3−phenylpropanoate degradation
glycogen degradation II (eukaryotic)
S−methyl−5−thio−α−D−ribo...hate degradation
L−methionine salvage cycle III
superpathway of tetrahydrofola...esis and salvage
methyl ketone biosynthesis
methanogenesis from acetate
L−leucine degradation I
catechol degradation I (meta−c...leavage pathway)
4−hydroxyphenylacetate degradation
chlorosalicylate degradation
mono−trans, poly−cis decapreny...ate biosynthesis
L−histidine degradation I
glycogen biosynthesis I (from ...m ADP−D−Glucose)
creatinine degradation I
catechol degradation to 2−oxop...pent−4−enoate II
catechol degradation II (meta−...leavage pathway)
benzoyl−CoA degradation I (aerobic)
L−ornithine degradation II (St...ckland reaction)
mycolyl−arabinogalactan−peptid...lex biosynthesis
superpathway of N−acetylglucos...nate degradation
superpathway of 2,3−butanediol...iol biosynthesis
superpathway of (R,R)−butanedi...iol biosynthesis
peptidoglycan biosynthesis V (...ctam resistance)
cob(II)yrinate a,c−diamide bio...obalt insertion)
0.0000
0.0025
0.0050
0.0075
Mean proportions
−0.004
−0.003
−0.002
−0.001
0.000
Difference in mean proportions
p−value(wilcox test)
4.112554e−02
4.112554e−02
4.112554e−02
4.112554e−02
4.112554e−02
4.112554e−02
3.794147e−02
3.408693e−02
3.408693e−02
2.597403e−02
2.597403e−02
2.597403e−02
2.597403e−02
2.597403e−02
2.597403e−02
1.937338e−02
1.515152e−02
1.515152e−02
1.515152e−02
1.515152e−02
1.515152e−02
1.515152e−02
1.006456e−02
8.658009e−03
8.127043e−03
4.329004e−03
4.329004e−03
4.329004e−03
4.329004e−03
4.329004e−03
